# The Proapoptotic Protein BNIP3 Interacts with VDAC to Induce Mitochondrial Release of Endonuclease G

**DOI:** 10.1371/journal.pone.0113642

**Published:** 2014-12-01

**Authors:** Xiaosha Zhang, Xiuwu Bian, Jiming Kong

**Affiliations:** 1 Institute of Pathology and Southwest Cancer Center, The Third Military Medical University, Chongqing, China; 2 Department of Human Anatomy and Cell Science, University of Manitoba, Winnipeg, Manitoba, Canada; University of Pittsburgh, United States of America

## Abstract

BNIP3 is a proapoptotic protein that induces cell death through a mitochondria-mediated pathway. We reported previously that mitochondrial localization of BNIP3 and translocation of EndoG from mitochondria to the nucleus are critical steps of the BNIP3 pathway. It is not clear, however, that how BNIP3 interacts with mitochondria. Here we show that expression of BNIP3 resulted in mitochondrial release and nuclear translocation of EndoG. Incubation of a recombinant GST-BNIP3 protein with freshly isolated mitochondria led to the integration of BNIP3 into mitochondria, reduction in the levels of EndoG in mitochondria and the presence of EndoG in the supernatant that was able to cleave chromatin DNA. Co-immunoprecipitation and mass spectrometry analysis reveals that BNIP3 interacted with the voltage-dependent anion channel (VDAC) to increase opening probabilities of mitochondrial permeability transition (PT) pores and induce mitochondrial release of EndoG. Blocking VDAC with a VDAC antibody largely abolished mitochondrial localization of BNIP3 and prevented EndoG release. Together, the data identify VDAC as an interacting partner of BNIP3 and support endonuclease G as a mediator of the BNIP3 pathway.

## Introduction

BNIP3 (Bcl-2/adenovirus E1B 19 kDa protein-interacting protein 3, also known as NIP3) is a member of a Bcl-2 subfamily of death-inducing mitochondrial proteins [1]. Due to a functional HIF-1-responsive element (HRE), BNIP3 is highly expressed in hypoxic and ischemic conditions [2] and has been shown to play a role in the pathogenesis of many neurodegenerative and cardiovascular diseases [3]. Loss of BNIP3 expression contributes to chemoresistance of cancer cells [4]. This 194-amino acid protein has 4 domains: a PEST domain that targets BNIP3 for degradation, a putative Bcl-2 homology 3 (BH3) domain that is homologous to other members of the Bcl-2 family, a conserved CD domain and a C-terminal transmembrane domain (TM). Unlike other members of the Bcl-2 family, the TM domain rather than the BH3 domain in BNIP3 is required for its dimerization, mitochondrial localization and death-inducing activities [5], [6]. BNIP3-induced cell death is characterized by rapid opening of the mitochondrial permeability transition (PT) pores, profound mitochondrial dysfunction and chromatin DNA cleavage but appears to be independent of caspase activity [6], [7]. We previously reported that the BNIP3 pathway involves mitochondrial release and nuclear translocation of the endonuclease G (EndoG) [8], [9]. It is not clear, however, that how BNIP3 interacts with mitochondria. Here we show that BNIP3 interacts with the voltage-dependent anion channel (VDAC) to directly induce mitochondrial release and nuclear translocation of EndoG. Our data identify VDAC as an interacting partner of BNIP3 and provide direct evidence to support that EndoG is a mediator of the BNIP3 cell death pathway.

## Materials and Methods

### Cell transfection and immunocytochemistry

SH-SY5Y human neuroblastoma cells were maintained in Dulbecco’s modified Eagle’s/F-12 (1∶1) medium supplemented with 100 units/ml penicillin, 100 µg/ml streptomycin and 10% fetal bovine serum. After 24 h in culture, the cells were transiently transfected with a pcDNA3-hBNIP3 plasmid that carried the full length of the human BNIP3 gene with LipofectAMINE 2000 (Invitrogen, Burlington, Ontario). Cells transfected with a pcDNA3-hBNIP3ΔTM plasmid carrying a dysfunctional form of BNIP3 due to deletion of the transmembrane domain were used as controls. Both plasmids were gifts from the late Dr. A.H. Greenberg [10]. Twenty-four hours after transfection, the cells were harvested for immunocytochemistry as described previously [9], [11].

### Production of recombinant proteins

Rat BNIP3 and BNIP3ΔTM cDNAs were obtained by RT-PCR using the following primers: sense 5′ –GGATCCATGTCGCAGAGCGGGGAGGA- 3′ for both BNIP3 and BNIP3ΔTM, and antisense 5′ –GAATTCTCAAAAGGTACTACTAGTGGAA for BNIP3 and 5′-GAATTCTAACAGAGATGGAAGGAAAA-3′ for BNIP3ΔTM. The RT-PCR products were cloned into pGEM-T vector (Promega) following the manufacturer’s protocol. After digestion of the recombinant plasmid with BamHI and EcoRI, the resulting fragments were purified and inserted into BamHI-EcoRI-digested expression vector pGEX-2T (Amersham Pharmacia Biotech) to generate the recombinant BNIP3 and BNIP3ΔTM plasmids. Recombinant GST-Bcl-2, GST-hBid and GST-tBid plasmids were generous gifts from Dr. Kaina [12], Dr. Yuan [13] and Dr. Korsmeyer [14] respectively. All constructs were confirmed by DNA sequencing. Then, 100 ml fresh LB culture medium was inoculated with 1.0 ml of BL21DE3 of each bacterial transfectant and vigorously shaken (180 rpm) at 37°C until the culture reached exponential phase (OD 0.5–0.8, about 3 hours). To induce the production of the fusion proteins, Isopropyl-b-D-thiogalactopyranoside (IPTG, 1 mM final concentration) was added to the BL21DE3. The bacterial cultures were pelleted and lysed with Bacterial Protein Extraction Reagent (B-PER, Pierce, Rockford, IL). The expressed proteins were recovered in the soluble bacterial fraction and purified by Glutatione Sepharose 4B (Amersham Biosciences) according to the manufacturer’s protocol.

### Isolation of mitochondria and incubation with recombinant proteins

Adult Sprague Dawley rats were deeply anesthetized with a mixture of isoflurane/propylene glycol (1 ml of the mixture per 500 ml of bell jar space, University of Manitoba animal care SOP A003). All procedures performed were approved by the Animal Care Committee at the University of Manitoba, whose standards meet the guidelines of the Canadian Council on Animal Care (CCAC). Fresh liver tissue was then obtained and homogenized in ice-cold isolation buffer containing 225 mM mannitol, 75 mM sucrose, 5 mM Hepes, and 1 mg/ml bovine serum albumin (pH 7.4). After centrifugation at 600 g for 10 min at 4°C to remove intact cells and nuclei, crude mitochondria were pelleted from the supernatant by centrifugation at 12,000 g for 10 min at 4°C and further purified on a Percoll gradient as described previously [15]. The mitochondrial pellet was resuspended in cell free system (CFS) buffer(10 mM HEPES-NaOH, pH 7.4, 220 mM mannitol, 68 mM sucrose, 2 mM NaCl, 2.5 mM KH_2_PO_4_, 0.5 mM EGTA, 2 mM MgCl_2_, 5 mM pyruvate, 0.1 mM PMSF, 1 mM dithiothreitol), kept on ice and used within 1 h of preparation. The mitochondria equivalent of 100 µg proteins were incubated at 37°C in 100 µl CFS buffer for 60 min with various recombinant proteins. The samples were separated into mitochondrial and supernatant fractions by centrifugation at 20,000 g for 10 min at 4°C for further analysis.

### Co-immunoprecipitation

After incubation with or without GST-BNIP3, mitochondria were resuspended in 1 ml nondenaturing lysis buffer (1% Triton X-100, 50 mM Tris-Cl, 300 mM NaCl, 5 mM EDTA add 10 mM iodoacetamide, 1 mM PMSF, 2 µg/ml leupetpin, pH 7.4) on ice for 30 min and centrifuged at 12,000 g for 10 min at 4°C. In experiments with VDAC antibody blocking, 0.3 mg/ml anti-VDAC/Porin antibody (Abcam, ab34726) was added to the mitochondrial samples 30 minutes before GST-BNIP3. The supernatants were incubated with a monoclonal BNIP3 antibody (1∶1000, University of Manitoba) or a VDAC antibody (1∶500, Cell Signaling, Danvers, MA) with shaking for 2 hrs on ice. Then, 150 µl Sepharose 4B was added to each sample to incubate for overnight at 4°C with gentle agitation. Immune complexes were precipitated with protein A agarose beads (Invitrogen). Followed by washes in 1 ml nondenaturing lysis buffer for three times, the pellet was resuspended in sample buffer, and proteins were resolved by SDS-PAGE (10% gel) and analyzed by Mass spectrometry and Western blotting with antibodies to BNIP3 (1∶1000) and VDAC (1∶500, Cell Signaling, Danvers, MA), as described previously [5]. Western blot bands were quantified using the NIH ImageJ software.

### Protein identification by Mass spectrometry

After Coomassie Brilliant Blue staining, protein bands in SDS-PAGE gels were excised and in gel digested with trypsin. Following centrifugation, the supernatants containing the tryptic peptides were used for Mass spectrometry, performed by the Manitoba Centre for Proteomics and Systems Biology, Winnipeg, Canada.

### Isolation of HEK293 nuclei and measurement of DNA fragmentation

Exponentially growing HEK293 cells were collected by centrifugation for 5 min at 200 g. Cells were washed in cold PBS and rocked on ice for 20 min in NB buffer (10 mM HEPES-NaOH pH 7.5, 10 mM KCl, 1.5 mM MgCl_2_, 1 mM DTT, 0.1 mM PMSF). Cells were dounced 20 times and the homogenate was mixed with a double volume of a sucrose cushion in NB complete buffer (NB+30% sucrose) and spun down at 500 g for 6 min at 4°C. The nuclear pellet was washed and resuspended in CFS buffer. For studying DNA fragmentation, 10^6^ HEK293 nuclei in 5 µl CFS buffer were mixed with 25 µl of the mitochondrial supernatant and incubated for 2 h at 37°C. The nuclei were then lysed in TE buffer (50 mM Tris-HCl pH 8.0, 10 mM EDTA) with 0.5% SDS and 0.5 mg/ml proteinase K for 4 h at 37°C. Proteins were extracted with TE-buffered phenol and DNA was precipitated with two volumes of EtOH and 1/10 volume of 4 M NaCl by overnight incubation at 72°C. The DNA samples were resuspended in TE and loaded on a horizontal 1.8% agarose gel.

### Detection of calcein release from mitochondria

Mitochondria (100 µg) suspended in 100 µl buffer containing (2 mM HEPES, pH 7.5, 0.25 M sucrose, 10 mM succinate, 1 mM potassium phosphate) were incubated with various recombinant proteins for 30 min at 37°C. Then, Calcein-AM (Molecular probes, final concentration 5 µM) was added and incubated for another 30 min. After washing, the fluorescence intensities of calcein were measured on a VICTOR^3^ 1420 Mulitlabel microplate reader (Perkin Elmer Life Sciences) at an excitation wavelength of 485 nm and an emission wavelength of 525 nm. The release of calcein was calculated as the percentage of calcein that escaped from mitochondria.

## Results

### BNIP3 induces EndoG translocation

**Figure 1:**
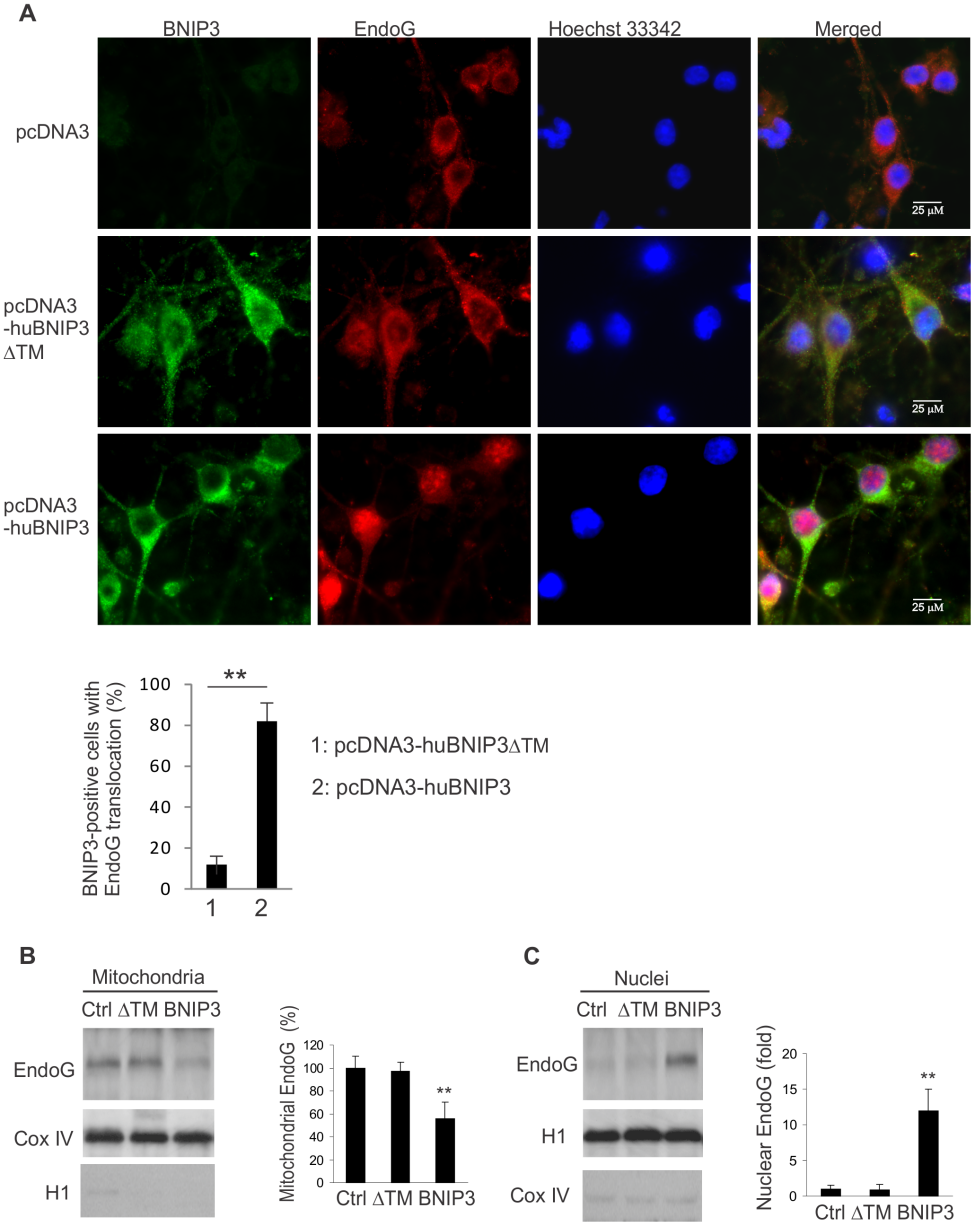
Expression of BNIP3 results in EndoG translocation. A, SH-SY5Y human neuroblastoma cells cells transfected with the full length BNIP3 or the mutant BNIP3ΔTM plasmids were immune-labeled with antibodies to BNIP3 and EndoG and counterstained with Hoechst 33342. Majority of BNIP3 expressing cells (82%) showed nuclear translocation of EndoG, while only 12% of cells expressing BNIP3ΔTM showed EndoG translocation. B and C, Western blot analysis of EndoG in mitochondrial and nuclear samples prepared from SH-SY5Y cells transfected with the indicated plasmids. Results shown represent the mean ±SD for combined data from 4 independent experiments. **, p<0.01.

To test whether expression of BNIP3 induces EndoG translocation, SH-SY5Y human neuroblastoma cells were transfected with a pcDNA3-huBNIP3 plasmid encoding the full length of human BNIP3. Immunohistochemical analysis showed that the majority (82%) of BNIP3 expressing cells had nuclear presence of EndoG (Figure 1A). In control experiments, EndoG translocation was detected in 12% of cells transfected with the truncated form of BNIP3 (pcDNA3-huBNIP3ΔTM). The BNIP3-induced EndoG translocation was further confirmed by Western blotting (Figure 1 B and C). In these experiments, mitochondria and nuclei were isolated from the cells 24 hours after transfection with the pcDNA3-huBNIP3 or pcDNA3-huBNIP3ΔTM plasmids and immuno-blotted with an EndoG antibody. As shown in Figure 1B, levels of EndoG in the mitochondria of BNIP3-expressing cells were significantly reduced (p<0.01) as compared to both the control and cells expressing the BNIP3ΔTM. Meanwhile, levels of EndoG in the nuclei were increased by 12 folds in the BNIP3-transfected cells (Figure 1C, p<0.01). Only background levels of nuclear EndoG were detected in the BNIP3ΔTM-expressing cells.

### BNIP3 induces mitochondrial release of EndoG in isolated mitochondria

**Figure 2:**
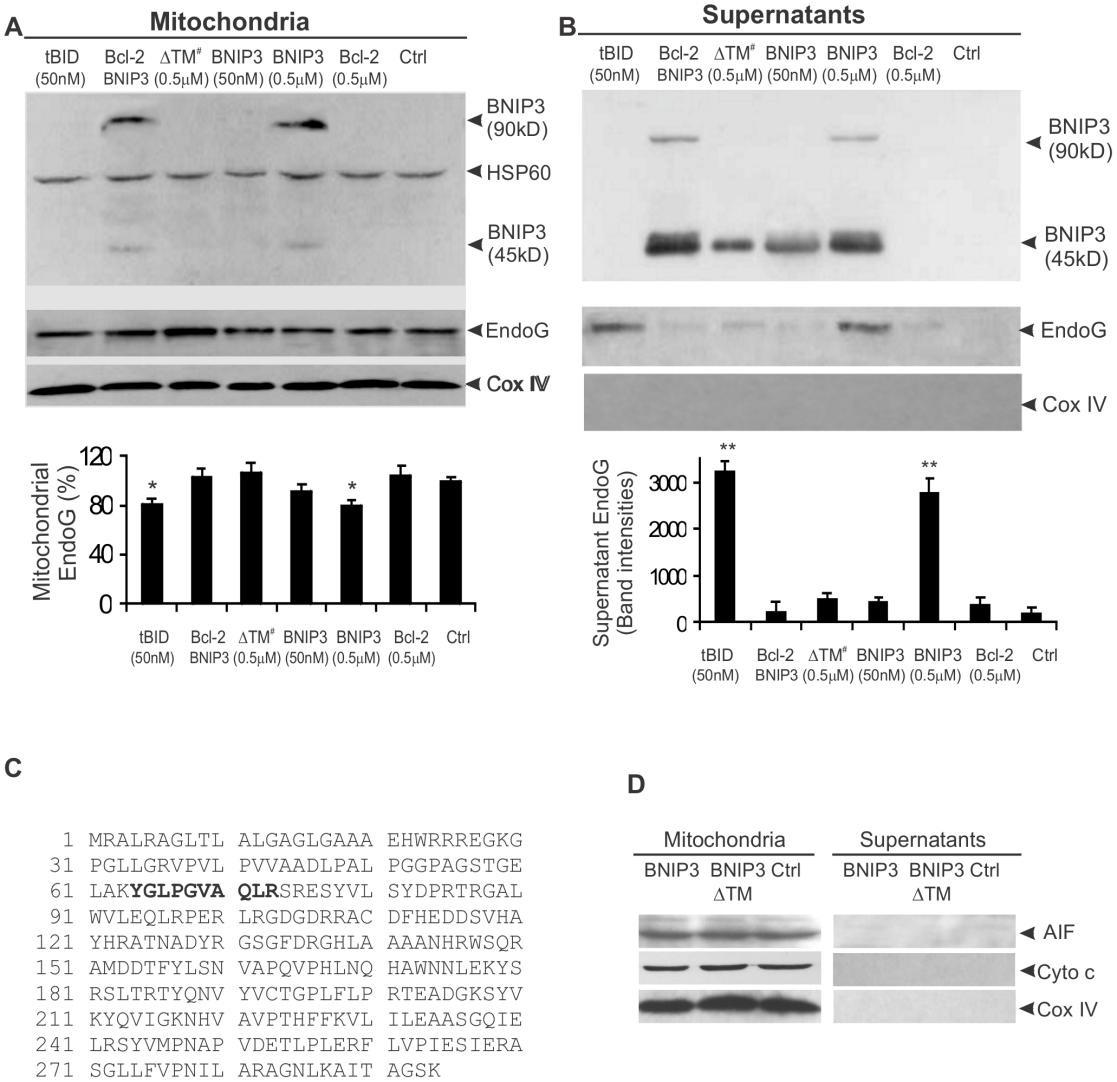
BNIP3-induced mitochondrial release of EndoG. Freshly isolated mitochondria were incubated with the indicated recombinant proteins for 1 h and then separated into mitochondrial and supernatant fractions. In the group treated with both Bcl-2 and BNIP3, both were used at 0.5 µM. A and B, Western blotting analysis of BNIP3 and EndoG in the mitochondrial and supernatant fractions. Loading controls were performed with Cox IV and HSP60 antibodies. Results shown represent the mean ±SD for combined data from three independent experiments. ΔTM ^#^, = BNIP3ΔTM; *, p<0.05; **, p<0.01. C, Amino acid sequence of EndoG. The tryptic peptide identified from the supernatant samples by mass fingerprinting is indicated in bold and underlined. D, Western blot analysis of cytochrome *c* and AIF. Both were not detectable in the supernatant samples.

Due to complexity of cellular events, the translocation of EndoG in BNIP3-expressing cells may be caused by factors indirectly or independently of BNIP3. To rule out this possibility, we took an in vitro approach using isolated mitochondria. We generated recombinant GST-BNIP3, GST-BNIP3ΔTM, GST-Bcl-2 and GST-tBid proteins, and isolated mitochondria from rat livers. Because calcium was known to be a factor in causing mitochondrial permeability transition (MPT) [16], we incubated freshly isolated mitochondria with the recombinant proteins for 1 hour at 37°C in calcium-free buffer as described previously [17]. Then, the samples were separated into mitochondrial and supernatant fractions and Western-blotted with antibodies to EndoG, BNIP3, AIF, Cox IV and HSP60. As expected, active GST-BNIP3 proteins, with a predicted molecular weight of about 45 kD, tended to form dimmers. The dimeric form of GST-BNIP3 (90 kD) was primarily found in the mitochondrial fraction (Figure 2A). Levels of EndoG in mitochondria were significantly reduced (p<0.05) by incubation with 0.5 µM GST-BNIP3. The truncated Bid (tBid), which is known to cause mitochondrial release of EndoG [17], was used as a positive control. There was a mild reduction of EndoG in mitochondria treated with 50nM GST-BNIP3. Meanwhile, high levels of EndoG were detected in the BNIP3- and tBid-treated mitochondrial supernatants (Figure 2B). In controls, EndoG release was not detected in mitochondria treated with Bcl-2 or with the inactive form of BNIP3 that lacked the functional transmembrane domain. When mitochondria were pre-incubated with Bcl-2 for 30 minutes before BNIP3, only background levels of EndoG were detected in the supernatant, suggesting that the BNIP3-induced mitochondrial release of EndoG could be inhibited by Bcl-2. To confirm the presence of EndoG in the BNIP3-treated mitochondrial supernatants, protein bands were excised and in-gel digested with trypsin followed by mass spectrometry. A tryptic peptide (YGLPGVAQLR) matching amino acids 64 to 73 of the rat EndoG was identified (Figure 2C).

**Figure 3:**
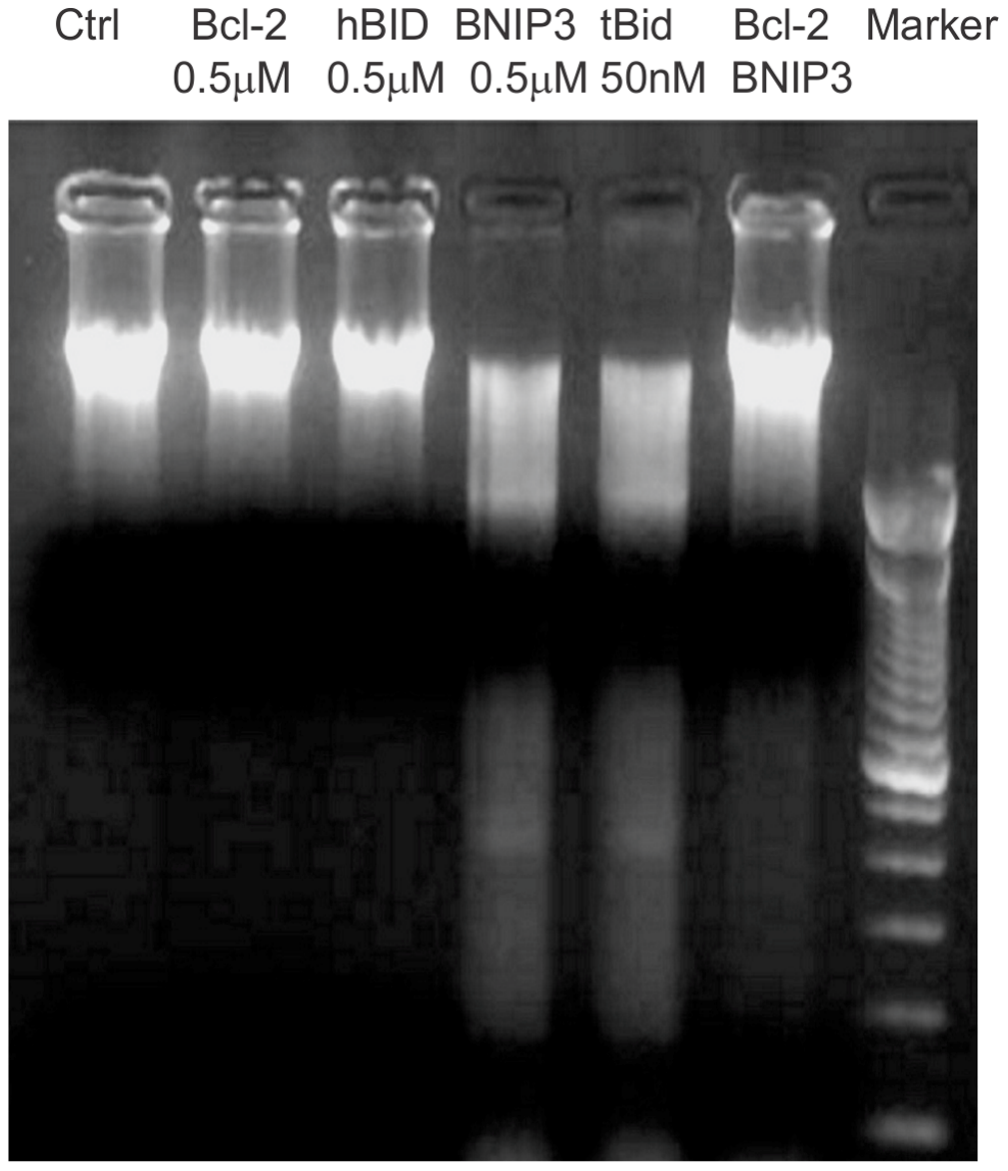
EndoG released from isolated mitochondria following BNIP3 induction is enzymatically active. Freshly isolated mitochondria were incubated with various recombinant proteins for 1 h at 37°C. The supernatants were collected and incubated with HEK293 nuclei for 2 h at 37°C. The DNA samples were loaded on a horizontal 1.8% agarose gel for DNA laddering assay. Shown is a representative image selected from three independent experiments.

To test the enzymatic activity of released EndoG, freshly isolated nuclei were incubated with the mitochondrial supernatants. DNA laddering assay showed that cleavage of chromatin DNA was detected in nuclear samples incubated with supernatants prepared from GST-BNIP3- and GST-tBid-treated mitochondria, but not from Bcl-2- and hBid (the full length human Bid which is inactive)-treated mitochondria and the non-treated control (Figure 3). Pre-treatment of mitochondria with Bcl-2 largely abolished the nuclease activity of BNIP3-treated mitochondrial supernatant. This is in accordance with the results that endonuclease G release was prevented in mitochondria pretreated with Bcl-2 (Figure 2).

### BNIP3 induces opening of mitochondrial permeability transition (PT) pores by interacting with VDAC

**Figure 4:**
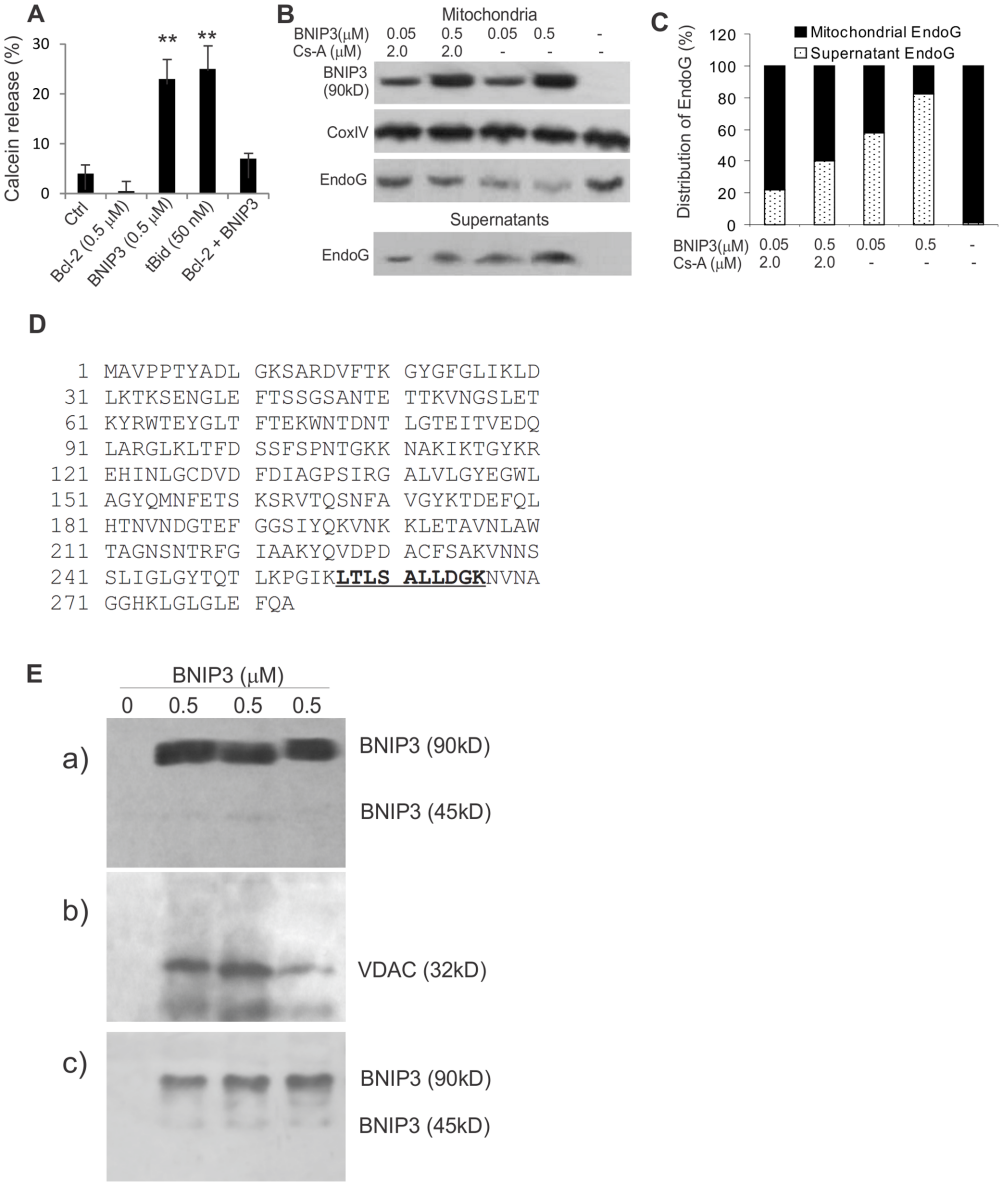
BNIP3 induces MPT by interacting with VDAC. A, BNIP3-induced MPT. Calcein release was presented as the percentage of calcein escaped from mitochondria during the 30-minute incubation. **, p<0.01 when compared with the control. B, Inhibition of PT pores reduced BNIP3-induced EndoG release. Cyclosporine A (2 µM) was added to mitochondria in CFS buffer 10 min before the BNIP3-GST protein at different final concentrations as indicated. BNIP3, CoxIV and EndoG in mitochondrial and supernatant fractions were detected by Western blotting. C, Distribution of EndoG was determined by analyzing the total intensities of the EndoG bands. D, Identification of VDAC as an interacting partner of BNIP3. After mitochondria-BNIP3 incubation, interacting proteins of BNIP3 were precipitated with a BNIP3 antibody and analyzed by Mass spectrometry fingerprinting. The amino acid sequence of VDAC with the tryptic peptide identified from the immunoprecipitates is labelled in bold and underlined. E Western bloting analyses show interaction between BNIP3 and VDAC. a) Levels of GST-BNIP3 in the mitochondrial fraction after mitochondria-BNIP3 incubation as determined by Western blotting. b) After co-immunoprecipitation with a BNIP3 antibody, immunoblotting with a VDAC antibody reveals the presence of VDAC in the BNIP3 immunoprecipitates. c) BNIP3 was present in the precipitates when a VDAC antibody was used for co-immunoprecipitation. Shown are samples from three independent experiments. Cell lysates without BNIP3 treatment were used as controls.

To test the effects of BNIP3 on mitochondrial PT pores, mitochondrial release of calcein was measured. The calcein-AM ester is a membrane-permeating fluorescent probe that freely enters mitochondria, but cannot exit except through an open PT pore following metabolism by esterases [18]. Significant calcein release was found in BNIP3- and tBid-treated mitochondria as compared with Bcl-2-treated and non-treated mitochondria (Figure 4A). Pre-treatment with Bcl-2 markedly reduced BNIP3-induced calcein release.

Members of the Bcl-2 family have been shown to interact with components of mitochondrial PT pores to induce mitochondrial dysfunction and selective release of mitochondrial proteins [19], [20]. To determine the involvement of PT pores in BNIP3-induced EndoG release, cyclosporine A, an inhibitor for mitochondrial PT pores, was added to the incubation 10 min before the BNIP3-GST. Inhibition of PT pores reduced BNIP3-induced EndoG release by 60% after 1 h of incubation (Figure 4, B and C). The result clearly suggests that the EndoG release is at least primarily through PT pores. To understand how BNIP3 interacts with mitochondria, we incubated mitochondria with GST-BNIP3 protein and performed co-immunoprecipitation experiments using our purified monoclonal BNIP3 antibody to pull down BNIP3-interacting proteins. By mass spectrometry, we identified a tryptic peptide that matches the amino acids 257 to 266 of the voltage-dependent anion channel (VDAC) (Figure 4D). The presence of VDAC in the BNIP3-interacting proteins was further confirmed by Western blotting using a specific VDAC antibody (Figure 4E, b). As expected, BNIP3 was detected in the precipitates when a VDAC antibody was used for the co-immunoprecipitation (Figure 4E, c).

### Blocking VDAC with an anti-VDAC antibody prevents BNIP3-induced EndoG release

**Figure 5:**
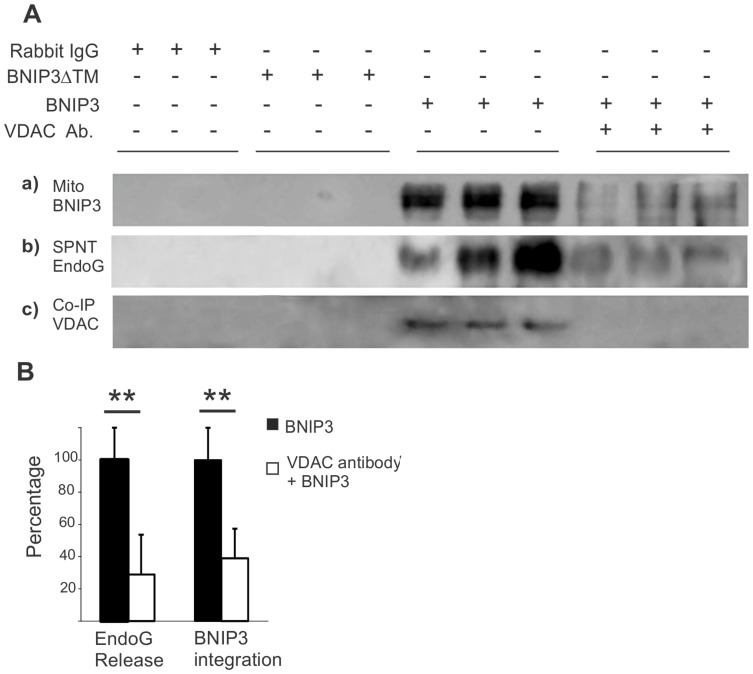
Blocking VDAC abolishes BNIP3-induced mitochondrial release of EndoG. A, Mitochondria (1 mg/ml) were incubated with a recombinant GST-BNIP3 in the presence of 0.3 mg/ml of an anti-VDAC antibody or normal rabbit IgG in control. Incubation with the trunked BNIP3 (BNIP3ΔTM) was used as a control. The samples were separated into mitochondrial and supernatant fractions by centrifugation. a), Blocking VDAC prevented BNIP3 from integration into mitochondria. b) Analysis of EndoG from the supernatant showed that levels of EndoG released from mitochondria was reduced by the VDAC antibody blocking. c) Co-immunoprecipitation with a BNIP3 antibody to pull down BNIP3-interacting proteins showed that blocking VDAC abolished the integration of BNIP3 with mitochondria. B, Quantification of BNIP3 integration with mitochondria and mitochondrial release of EndoG affected by the VDAC antibody blocking. Results shown represent the mean ±SD for combined data from three independent experiments. **, p<0.01.

To test if the activity of VDAC is required for BNIP3-induced cell death, mitochondria (1 mg/ml) were incubated with 0.3 mg/ml of an anti-VDAC antibody (Abcam ab34726) or normal rabbit IgG in control for 30 min before the addition of GST-BNIP3. As shown in Figure 5, blocking VDAC with a neutralizing antibody abolished the integration of BNIP3 into mitochondria and significantly reduced EndoG release from mitochondria. Co-immunoprecipitation with our BNIP3 antibody further verified that the VDAC antibody did block interaction of BNIP3 with VDAC.

## Discussion

Characteristically in apoptosis, nucleosomal fragmentation of DNA is a caspase-dependent event and results from activation of DNA fragmentation factor, a caspase-activated deoxyribonuclease [21]. BNIP3-induced cell death involves DNA cleavage but appears to be independent of caspase activity [7], [9], [22]. Therefore, other candidate nucleases are implicated. EndoG is one such nuclease that is able to cleave chromatin DNA independently of caspase activity [17]. It is implicated in diseases such as cardiac hypertrophy and Parkinson’s disease [23], [24]. EndoG is encoded in the nucleus, synthesized as a propeptide in the cytoplasm and imported into mitochondria through a process mediated by its amino-terminal mitochondrion-targeting sequence. This signal peptide is cleaved off upon entering mitochondria and thus mitochondrial EndoG is mature and active [25]. A previous study has demonstrated that Endo G is the predominant mitochondrial DNase because a neutralizing antibody to Endo G fully blocked DNA degradation of mitochondrial nucleases in vitro [17]. We previously observed the involvement of EndoG in BNIP3-induced cell death in models of hypoxia and cerebral ischemia [8], [9], [26]. In the present study, we found that forced expression of BNIP3 caused in mitochondrial release and nuclear translocation of EndoG. Incubation of a recombinant GST-BNIP3 protein with freshly isolated mitochondria led to the integration of BNIP3 into mitochondria, reduction in the levels of EndoG in mitochondria and the presence of EndoG in the mitochondrial supernatant that was able to cleave chromatin DNA. Although mitochondria contain another nuclease (endonuclease G-like 1, EXOG), the fact that the substrates we used were HEK293 nuclei argues that the nuclease activities we detected were primarily from EndoG because only EndoG cleaves double stranded DNA while endonuclease G-like 1 (EXOG) is known to cleave preferably single stranded DNA [27]. From these data, we conclude that BNIP3 directly induce mitochondrial release of EndoG. Interestingly, the mitochondrial proteins apoptosis inducing factor (AIF) and cytochrome c were not detected in both BNIP3 and BNIP3ΔTM-treated mitochondrial supernatants. This is in agreement with a previous observation in cell cultures [7], suggesting that BNIP3 may selectively induce mitochondrial release of EndoG and that AIF and cytochrome c may not be involved in the BNIP3 pathway.

Mitochondrial release of apoptotic proteins occurs often through the opening of the mitochondrial PT pores [28]. Calcium is a known important inducer of mitochondrial permeability transition (MPT). MPT can also be a calcium-independent process as demonstrated previously [17], [29]. Previous studies in cell cultures showed that expression of BNIP3 increased opening probability of mitochondrial PT pores [7]. In our experiments, we have observed that BNIP3 is able to induce MPT in calcium-free media, suggesting that BNIP3-induced MPT is calcium-independent. Our data also support that BNIP3-induced mitochondrial release of EndoG is calcium-independent and that BNIP3 induced MPT is different from calcium-induced MPT because the later has been shown to cause cytochrome *c* release [16].

One critical event of the BNIP3 cell death pathway is its interaction with mitochondria to induce mitochondrial release of proteins [7], [9], [10]. Unlike many death-inducing proteins BNIP3 does not have a functional “intracellular” domain, suggesting that BNIP3 needs an interacting partner for its death-inducing activity. By co-immunoprecipitation and mass spectrometry analysis we provide the first evidence that BNIP3 interacts with the voltage-dependent anion channel (VDAC) to increase opening probabilities of mitochondrial permeability transition (PT) pores and induce mitochondrial release of EndoG. Blocking VDAC with a VDAC antibody is able to abolish the mitochondrial localization of BNIP3 and prevent EndoG release. Together, the data suggest that VDAC is an interacting partner of BNIP3. Further pharmacological and genetic experiments are needed to demonstrate whether VDAC is functionally required for BNIP3-induced EndoG release.

In summary, the present study provides evidence that BNIP3 interacts with VDAC to induce mitochondrial release of EndoG. The results identify VDAC as an interacting partner of BNIP3 and support that EndoG is a mediator of the BNIP3-induced cell death pathway. Further experiments with EndoG knockout animals or cells will determine whether EndoG is functionally required for the BNIP3 pathway.
